# Rat Hepatitis E Virus in Norway Rats, Ontario, Canada, 2018–2021

**DOI:** 10.3201/eid2909.230517

**Published:** 2023-09

**Authors:** Sarah J. Robinson, Jamie Borlang, Chelsea G. Himsworth, David L. Pearl, J. Scott Weese, Antonia Dibernardo, Carla Osiowy, Neda Nasheri, Claire M. Jardine

**Affiliations:** University of Guelph, Guelph, Ontario, Canada (S.J. Robinson, D.L. Pearl, J.S. Weese, C.M. Jardine);; Public Health Agency of Canada, Winnipeg, Manitoba, Canada (J. Borlang, A. Dibernardo, C. Osiowy);; University of British Columbia, Vancouver, British Columbia, Canada (C.G. Himsworth);; Health Canada, Ottawa, Ontario, Canada (N. Nasheri);; University of Ottawa, Ottawa (N. Nasheri);; Canadian Wildlife Health Cooperative, Guelph (C.M. Jardine)

**Keywords:** hepatitis E virus, Norway rats, *Rattus norvegicus*, *Rocahepevirus ratti*, rats, public health, zoonoses, Canada

## Abstract

We tested liver samples from 372 Norway rats (*Rattus norvegicus*) from southern Ontario, Canada, during 2018–2021 to investigate presence of hepatitis E virus infection. Overall, 21 (5.6%) rats tested positive for the virus. Sequence analysis demonstrated all infections to be rat hepatitis E virus (*Rocahepevirus ratti* genotype C1).

Hepatitis E virus (HEV) is a nonenveloped, single-stranded, positive-sense RNA virus within the family Hepeviridae, subfamily Orthohepevirinae, which is divided into 4 genera: *Paslahepevirus*, *Rocahepevirus*, *Chirohepevirus*, and *Avihepevirus* (*1*). Human hepatitis E is primarily caused by *Paslahepevirus balayani* (genotypes 1–4), and infection generally causes an acute, self-limiting disease, but severe and chronic hepatitis and extrahepatic manifestations can occur in immunocompromised patients (*2*). *Paslahepevirus balayani* genotypes 1 and 2 are endemic in developing countries, circulating in humans and transmitted primarily via the fecal-oral route through contaminated drinking water. Sporadic cases of hepatitis E infection caused by zoonotic transmission of HEV (*Paslahepevirus balayani* genotypes 3 and 4) are increasingly reported in industrialized countries (*3*). Infections are acquired through direct contact with infected animals, environmental contamination with animal feces, and foodborne transmission from eating undercooked pork, venison, and wild boar meat (*3*).

Additional HEV variants have been reported in a diversity of animal species, and zoonotic transmission from animal reservoirs is a growing public health concern. Norway rats (*Rattus norvegicus*) have been shown to carry swine HEV (*Paslahepevirus balayani* genotype 3) and are natural reservoirs of HEV variants within the species *Rocahepevirus ratti* genotype C1 (rat HEV) (*4*). Since it was first detected in Germany in 2010, rat HEV has been identified in Norway rats from the United States, China, Vietnam, and 13 countries in Europe (*5*). Recently, cases of acute hepatitis caused by rat HEV have been reported in Hong Kong, Canada (infection acquired in Central Africa), and Spain (*6*–*8*). Those reports raise concerns regarding the potential risk for rat HEV transmission to humans and hepatitis E as an emerging infectious disease worldwide.

## The Study

**Figure 1:**
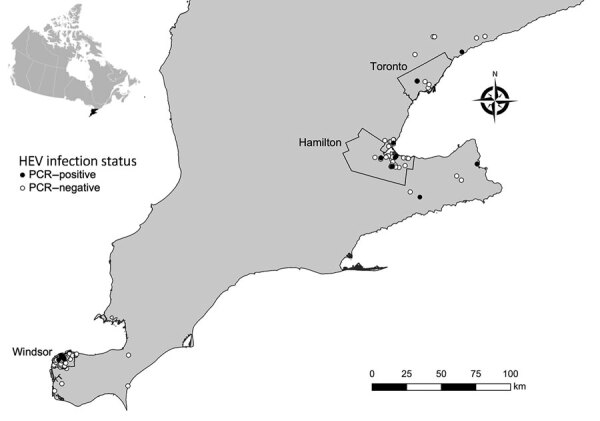
Sites of collection and HEV PCR status of rats submitted by pest control professionals in southern Ontario, Canada, during November 2018–June 2021. Geographic administrative boundaries of the cities of Windsor, Hamilton, and Toronto are displayed. Inset map indicates the location of southern Ontario within Canada. HEV, hepatitis E virus.

We conducted a study to investigate HEV infection in Norway rats from southern Ontario, Canada, and identify associations between host factors, season, land use, and year of collection. We obtained rat carcasses through collaboration with pest control professionals working in southern Ontario. Our rat and sample collection methods have been previously described and evaluated as a source of samples for zoonotic pathogen surveillance (*9*). We studied 372 Norway rats (species determined by external morphology) from 161 unique geographic coordinates within southern Ontario during November 2018–June 2021 (Figure 1). During necropsy, we recorded rat demographic characteristic data (Table) and collected liver samples aseptically. Most rats in our sample were sexually mature (65%), and there were more females (51.2%) than males (48.8%). We noted the body condition of rats to be poor (emaciated or underconditioned) in 69.1% and good (well conditioned or overconditioned) in 30.9%. We could not determine sex (3%), sexual maturity (3.2%), or body condition (2.4%) in a minority of rats because of poor carcass condition. We categorized rats by collection location as residential (52.4%), industrial (17.5%), institutional (15.6%), commercial (8.9%), and mixed (5.6%) land use. We collected most rats during the winter (36.8%) and fall (36.8%), followed by spring (21.8%) and summer (4.6%). To account for low sample size in the summer, we recategorized seasonal data as summer/fall (June–November) and winter/spring (December–May).

**Table:** Descriptive statistics for rat demographic variables, land use, season, and year of collection and results from exact logistic regression analyses evaluating associations with hepatitis E virus PCR status among 372 Norway rats (collected in southern Ontario, Canada, during November 2018–June 2021

Category, no. with data available	No. (%)	PCR-positive (%)	PCR-negative (%)	Odds ratio (95% CI)	p value
Sex, n = 361					
F	185 (51.2)	9 (4.9)	176 (95.1)	Referent	
M	176 (48.8)	11 (6.3)	165 (93.7)	1.13 (0.43–2.95)	0.955
Sexual maturity, n = 360					
Immature	126 (35.0)	1 (0.8)	125 (99.2)	Referent	
Mature	234 (65.0)	19 (8.1)	215 (91.9)	3.99 (1.14–21.47)	0.025
Body condition, n = 363					
Poor	251 (69.1)	11 (4.4)	240 (95.6)	Referent	
Good	112 (30.9)	9 (8.0)	103 (92.0)	1.66 (0.61–4.36)	0.361
Land use, n = 372*					
Residential	195 (52.4)	11 (5.6)	184 (94.4)	Referent	
Nonresidential	177 (47.6)	10 (5.6)	167 (94.4)	0.92 (0.35–2.39)	1.000
Season, n = 372†					
Summer/fall	154 (41.4)	8 (5.2)	146 (94.8)	Referent	
Winter/spring	218 (58.6)	13 (6.0)	205 (94.0)	1.03 (0.39–2.80)	1.000
Year of collection, n = 372					
2018	43 (11.6)	4 (9.3)	39 (90.7)	Referent	
2019	193 (51.9)	11 (5.7)	182 (94.3)	0.47 (0.14–1.84)	0.307
2020	93 (25.0)	2 (2.2)	91 (97.8)	0.17 (0.02–1.12)	0.069
2021	43 (11.6)	4 (9.3)	39 (90.7)	0.80 (0.15–4.04)	1.000

*Land use was defined as residential and nonresidential (i.e., institutional, industrial, commercial, and mixed). †Seasons were defined as winter (December–February), spring (March–May), summer (June–August), and fall (September–November).

We screened liver RNA extracts for the presence of HEV by real-time PCR by using previously described primers and probes (*10*). Of 372 rats tested, 21 (5.6%, 95% CI 3.5%–8.5%) rats from 16 distinct locations in 7 cities/towns were positive for HEV (Figure 1). The odds of HEV infection were significantly higher in sexually mature rats (odds ratio 3.99, 95% CI 1.14–21.47; p = 0.025). By using exact logistic regression models, we observed no association with sex, body condition, land use, season, or year of collection (Table).

**Figure 2:**
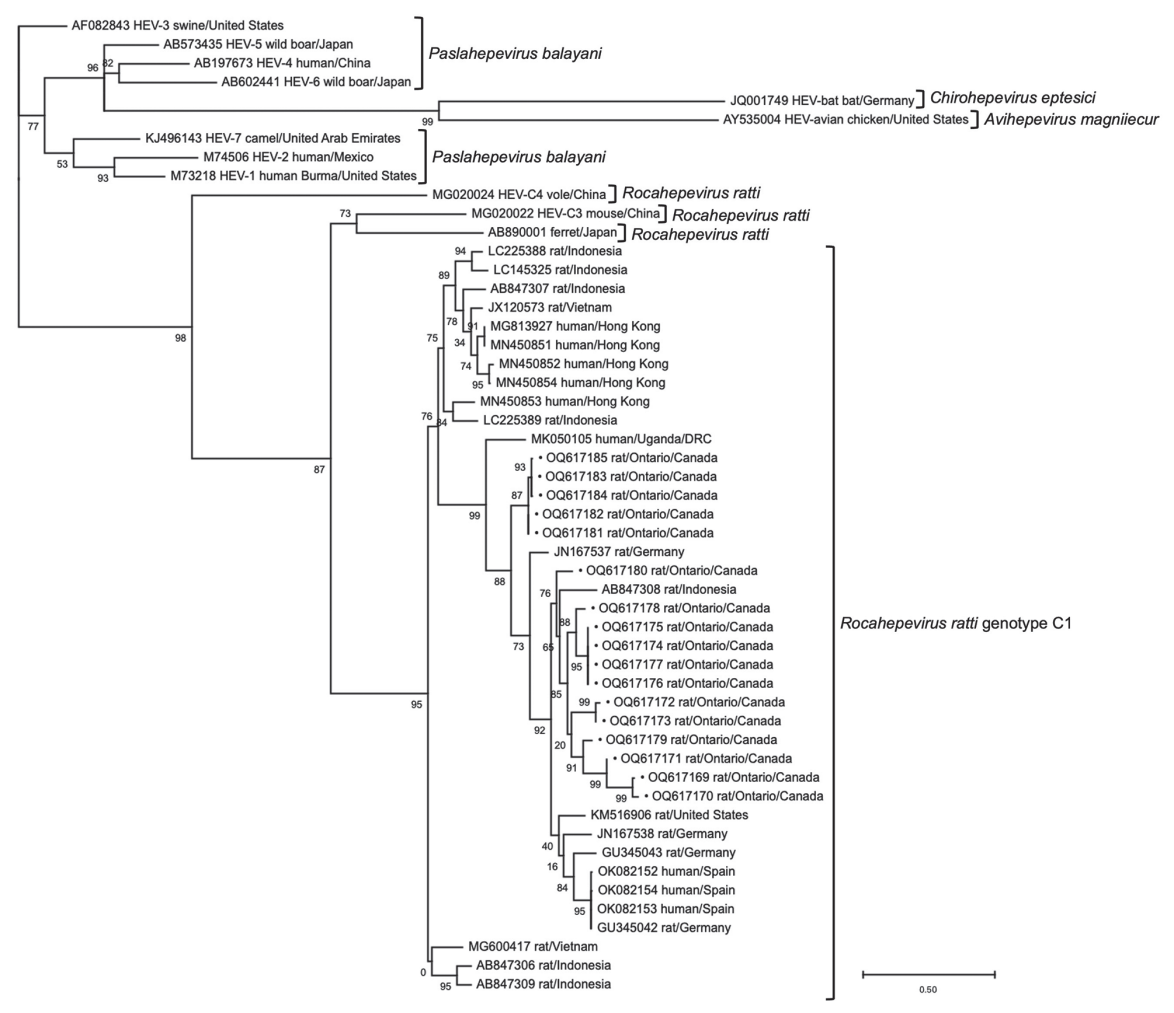
Phylogenetic tree based on the nucleotide alignment of hepatitis E virus (HEV) sequences from rats submitted by pest control professionals in southern Ontario, Canada, during November 2018–June 2021 (black dots), and select reference sequences from other studies in GenBank (accession numbers provided). Maximum-likelihood analysis of a 283-nt fragment of the RNA dependent RNA polymerase of open reading frame 1 was performed by the general time reversible plus gamma plus invariant sites substitution model as determined for the alignment by Smart Model Selection (*12*). Tree construction was optimized by nearest neighbor interchange and subtree pruning and regrafting with branch support computed by the approximate likelihood-ratio test based on a Shimodaira-Hasegawa-like procedure (*13*). Only bootstrap values >70% are shown. Scale bar indicates the number of nucleotide substitutions per site. DRC, Democratic Republic of the Congo.

We amplified positive samples by using a previously described heminested PCR to generate an amplicon from the open reading frame (ORF) 1 region (*11*). We retrieved a 283-nt fragment of ORF1 from 17 samples and analyzed generated sequences with Lasergene software (DNASTAR, https://www.dnastar.com). We did not obtain sequence data for 4 rats. We aligned sequences with select GenBank reference sequences representing HEV genotypes currently known to infect rats, as well as rat HEV found in humans. Phylogenetic analysis of the partial ORF1-derived sequences showed that all PCR amplicons were rat HEV. We grouped rat HEV sequences from southern Ontario (GenBank accession numbers OQ617169–85) into 4 distinct clusters (Figure 2), with relatively low genetic divergence (14%). Sequences in our study had the highest nucleotide homology with rat HEV sequences from rats in the United States (83.3%), followed by Germany (82.2%), Vietnam (71.5%), and Indonesia (71.3%). Southern Ontario shares a border with 2 US states, New York and Michigan, and the westernmost samples from Windsor were collected directly adjacent to Detroit, Michigan. We noted Ontario rat HEV sequences to be genetically distinct (24.6% divergence) from rat HEV sequences reported in humans.

## Conclusions

Laboratory analysis of samples taken from Norway rats in southern Ontario, Canada, revealed hepatitis E virus RNA in 21 (5.6%, 95% CI 3.5%–8.5%) of 372 rats, and phylogenetic analysis demonstrated that these sequences were closely related to those found in rats from other countries. Detection of rat HEV (*R. ratti* genotype C1) in Norway rats in our study shows that this virus is broadly distributed within southern Ontario, including 3 major cities (i.e., Toronto, Hamilton, and Windsor), and may be endemic in Norway rat populations. An absence of PCR-positive rats in some areas of southern Ontario may be the result of undersampling rather than an indication that HEV is absent in these populations.

We observed that sexually mature rats were at significantly greater odds of being infected with HEV than immature rats. This observation is in contrast to findings from previous studies of rats, which found no association with age and infection status (*14*,*15*). We concede that this disparity in findings might be owing to methodological differences in how age classes were defined (i.e., sexual maturity [open vaginal orifice in females, scrotal testes in males] vs. weight). The observed association in our study might be the result of cumulative exposure to HEV leading to increased risk for infection over time and behaviors in sexually mature rats that may increase transmission (e.g., exploratory and aggressive behaviors).

To date, 12 human cases of rat HEV have been reported in Hong Kong, Canada, and Spain (*6*–*8*). Although zoonotic transmission from rats to humans has been suggested, the exact source and route of transmission in these cases remains unclear. Notably, human hepatitis E caused by rat HEV may be underreported because of subclinical or mild infection, limited awareness, and diagnostic testing techniques for HEV that might not detect rat HEV. Further studies are needed to investigate potential modes and patterns of transmission and elucidate the zoonotic potential of rat HEV and associated public health risks.

This report of rat HEV (*R. ratti* genotype C1) in Canada provides further evidence that this virus has a broad geographic distribution globally and may be endemic in Norway rats. Our study highlights the importance of continued surveillance for HEV in rats and the need for additional research regarding the role of rats in human hepatitis E.
